# Asymmetric Au-catalyzed cycloisomerization of 1,6-enynes: An entry to bicyclo[4.1.0]heptene

**DOI:** 10.3762/bjoc.7.116

**Published:** 2011-07-26

**Authors:** Alexandre Pradal, Chung-Meng Chao, Patrick Y Toullec, Véronique Michelet

**Affiliations:** 1 Laboratoire Charles Friedel, UMR 7223, Ecole Nationale Supérieure de Chimie de Paris, Chimie ParisTech, 11 rue P. et M. Curie, F-75231 Paris Cedex 05, France

**Keywords:** asymmetric catalysis, bicycloheptene, cycloisomerization reactions, enynes, gold

## Abstract

A comprehensive study on the asymmetric gold-catalyzed cycloisomerization reaction of heteroatom tethered 1,6-enynes is described. The cycloisomerization reactions were conducted in the presence of the chiral cationic Au(I) catalyst consisting of (*R*)-4-MeO-3,5-(*t*-Bu)_2_-MeOBIPHEP-(AuCl)_2_ complex and silver salts (AgOTf or AgNTf_2_) in toluene under mild conditions to afford functionalized bicyclo[4.1.0]heptene derivatives. The reaction conditions were found to be highly substrate-dependent, the best results being obtained in the case of oxygen-tethered enynes. The formation of bicyclic derivatives, including cyclopropyl pentasubstituted ones, was reported in moderate to good yields and in enantiomeric excesses up to 99%.

## Introduction

**Scheme 1:**
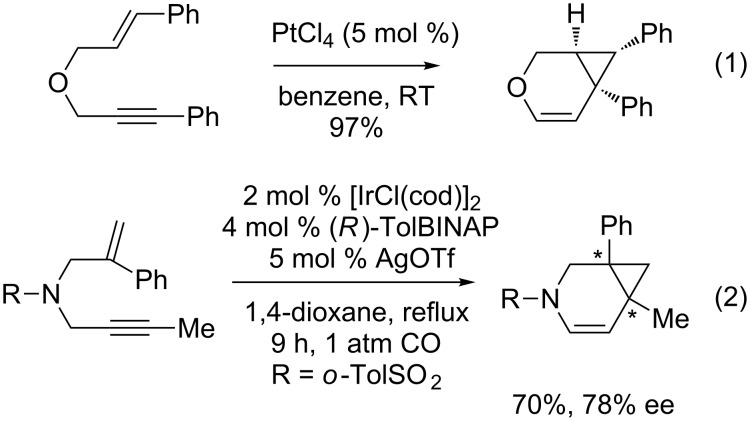
First reports on the racemic and asymmetric synthesis of bicyclo[4.1.0]heptenes.

Metal-catalyzed cycloisomerization reactions of 1,*n*-enynes have emerged as efficient processes that contribute to sustainable development and atom economy concepts [1–8]. In the last ten years, they have provided extremely efficient access to cyclic skeletons with a broad range of functional moieties. Among them, the synthesis of oxa- and azabicyclo[4.1.0]heptenes starting from heteroatom-linked 1,6-enynes has been recently a field of high interest considering the fundamental skeleton rearrangement research of 1,*n*-enynes [1–11] and the potential applications in biological active and natural products [12–13]. In 1995, Blum et al. described a novel PtCl_4_-catalyzed cycloisomerization reaction of allyl propynyl ethers leading to oxabicyclo[4.1.0]heptenes [14] (Scheme 1, reaction 1). The group of Murai observed a similar reactivity in the presence of PtCl_2_, although in a lower yield [15]. These seminal contributions were then followed by several comprehensive studies involving carbophilic complexes such as platinum or gold [16–22] that led to the formation of complex bicyclic and tricyclic compounds [23–40]. The first asymmetric version was described by Shibata’s group in 2005 in the presence of a chiral iridium catalyst [41] (Scheme 1, reaction 2). We and others recently pursued the improvement and development of this enantioselective process, by employing platinum [42–44], rhodium [45] or gold [46–48] complexes. Following our previous work with chiral gold catalysts [46], we report a comprehensive study on gold-catalyzed enantioselective synthesis of bicyclo[4.1.0]heptenes, focusing on the scope and limitations of such systems.

## Results and Discussion

### Optimization of the catalytic system

Based on our ongoing program on asymmetric gold catalysis [46,49–52], and on literature reports [53–55], we selected 4-MeO-3,5-(*t*-Bu)_2_-MeOBIPHEP-(AuCl)_2_ complex [56–58] as the best candidate for such a transformation. Initial experiments were performed using *N*-tosyl allyl substrate **1a** and oxygen-linked propargylic 1,6-enyne **2a** as model substrates (Table 1). The reaction of **1a** was evaluated in various solvents and proceeded smoothly leading to the desired alkene **3a** [59]. The reaction kinetics and stereoselectivity were found to be highly solvent-dependent, the enantiomeric excesses (ee) varying from 31% to 78% at room temperature (Table 1, entries 1–3). The reaction kinetic was very slow at room temperature in ether and toluene, but high ee’s were obtained. Increasing the temperature to 40 °C in toluene or ether had a positive effect both on the conversion and on the ee’s (Table 1, entries 4 and 5). The reaction was also conducted at 60 °C or 70 °C with good conversions and ee’s (Table 1, entries 6–8), the best results being obtained in toluene. At 80 °C in toluene, a decrease in the stereoselectivity was observed as the ee dropped to 91% (Table 1, entry 9). The reactivity of oxygen-tethered enynes such as **2a** was different to that for **1a** as a complete conversion was observed at room temperature in toluene, dichloromethane, ether and tetrahydrofuran (Table 1, entries 10–13). A better ee was obtained in toluene compared to other solvents. Cyclopropyl alkene **4a** was isolated in 56% yield and 96% ee at 0 °C in toluene (Table 1, entry 14). Toluene was therefore chosen for further studies.

**Table 1:** Cycloisomerization reaction of nitrogen- and oxygen-linked 1,6-enynes **1a** and **2a**.

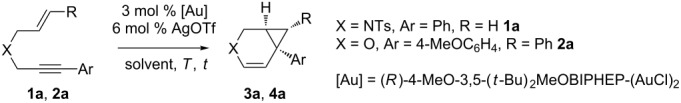							

Entry	Substrate	Solvent	*T* [°C]	*t* [h]	Conv. (Yield) [%]^a^	Product	ee [%]^b^

1	**1a**	CH_2_Cl_2_	RT	36	78	**3a**	31 (−)
2	**1a**	Et_2_O	RT	39	17	**3a**	75 (−)
3	**1a**	toluene	RT	39	11	**3a**	78 (−)
4	**1a**	Et_2_O	40	41	28	**3a**	90 (−)
5	**1a**	toluene	40	96	100 (47)	**3a**	98 (−)
6	**1a**	toluene	60	96	100 (74)	**3a**	98 (−)
7	**1a**	THF	60	96	69	**3a**	74 (−)
8	**1a**	toluene	70	96	100 (83)	**3a**	96 (−)
9	**1a**	toluene	80	48	66	**3a**	91 (−)
10	**2a**	toluene	RT	30	100 (57)	**4a**	92 (−)
11	**2a**	CH_2_Cl_2_	RT	25	100 (26)	**4a**	70 (−)
12	**2a**	Et_2_O	RT	25	100 (35)	**4a**	91 (−)
13	**2a**	THF	RT	25	100 (43)	**4a**	85 (−)
14	**2a**	toluene	0	120	100 (56)	**4a**	96 (−)

^a^Determined by ^1^H NMR, ^b^determined by HPLC.

### Synthesis of 1,6-enynes

**Scheme 2:**
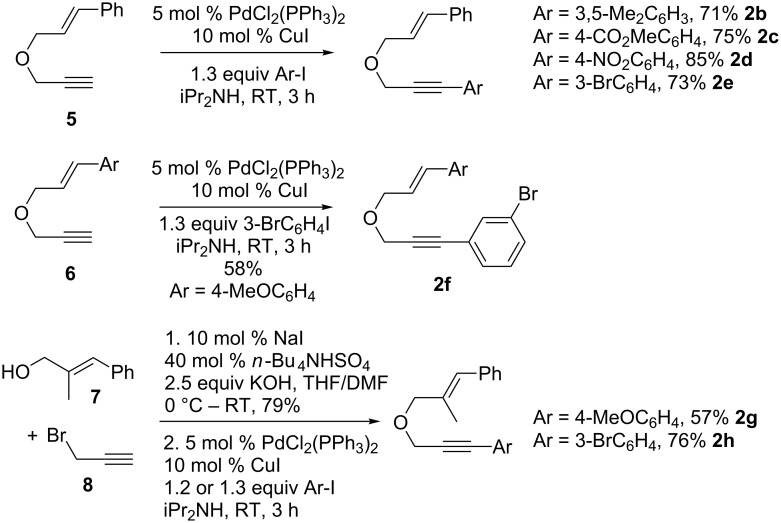
Synthesis of oxygen-tethered 1,6-enynes.

**Scheme 3:**
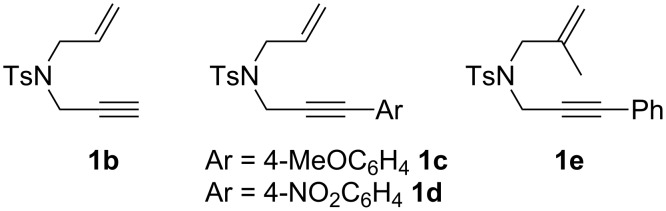
Nitrogen-tethered 1,6-enynes.

We prepared various oxygen-tethered 1,6-enynes according to classic methodologies employing a Williamson alkylation reaction and/or a Sonogashira cross-coupling [60–61] (Scheme 2 and Scheme 3). The known enyne **5** [62–63] was engaged in Pd-catalyzed coupling in the presence of diversely functionalized aryl iodides (Scheme 2). The corresponding substituted alkynes **2b–e** [46] were isolated in 71–85% yield. An analogous 1,6-enyne **6** [64] was also reacted with 3-bromoiodobenzene under the same reaction conditions and led to the formation of substrate **2f** in 58% isolated yield. We also envisaged preparing two trisubstituted alkenes **2g** and **2h** by an alkylation/Sonogashira sequence starting from commercially available substrates **7** and **8**.

We also selected some nitrogen-tethered 1,6-enynes **1b–e** from the literature [23,41–44] and synthesized them to evaluate the efficiency of the gold chiral catalytic system (Scheme 3).

### Scope and limitations of the catalytic system

The prepared heteroatom-linked 1,6-enynes were then engaged in the cycloisomerization process in the presence of Au(I) cationic catalyst generated by mixing (*R*)-4-MeO-3,5-(*t*-Bu)_2_-MeOBIPHEP-(AuCl)_2_ complex and silver salts (Table 2). Anticipating the moderate reactivity of nitrogen-tethered enynes **1**, the reactions were conducted at 60 °C in toluene (Table 2, entries 1–5). The substitution of the aromatic ring on the alkyne moiety led to a substantial decrease of both isolated yields and ee’s, as the presence of several by-products was detected, presumably due to degradation or polymerization [15]. A good ee was achieved in the case of enyne **1c**, by using AgNTf_2_ [65] instead of AgOTf (Table 2, entry 2 compared to entry 1). The substitution of the allylic side chain seemed to slow down the degradation process, as the cyclic alkene **3e** was isolated in 61% yield (Table 2, entry 4). In the case of non-substituted enyne **1b** (Table 2, entry 5), the bicyclic alkenyl derivative **3b** was isolated in low yield and ee: The synthesis of **3b** was accompanied by the formation of known 1,3- and 1,4-dienes (5% and 10% isolated yield respectively) resulting from 5-*exo*- and 6-*endo* cycloisomerization reactions [20,66]. Thus, the gold catalytic system cannot compete with the results obtained for the cyclizations of nitrogen-tethered enynes in the presence of iridium, platinum or rhodium catalysts [41–45]. The cycloisomerization process was found to be highly stereoselective in the case of oxygen-tethered enynes (Table 2, entries 6–12). In all cases, the ee’s were greater than 90% and in one case as high as 98%. The stability of the resulting bicyclic alkenes **4** was generally only moderate, which led to low isolated yields. In the case of 1,6-enynes **2c** and **2d**, the low yields (25% and 32% respectively) could be improved by switching from AgOTf salt to AgNTf_2_, presumably due to the experimentally observed lower hygroscopicity of bistriflimide complex (Table 2, entry 7 compared to 8 and 9 compared to 10). The functionalized derivatives **4c** and **4d** were obtained in 64% and 63% yields respectively and in excellent ee’s (Table 2, entries 8 and 10). The compatibility with another functional group on the aromatic ring such as bromine (Table 2, entry 11), and with a different allylic side chain (Table 2, entry 12) was also evaluated: The corresponding bicyclic adducts **4e** and **4f** were isolated in modest to good yield and 95% ee.

**Table 2:** Cycloisomerization reaction of nitrogen- and oxygen-linked 1,6-enynes.

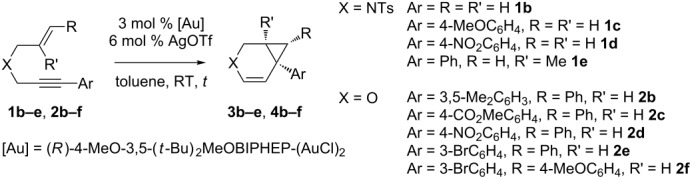							

Entry	Enyne		*t* [h]	Yield [%]^a^	Product		ee [%]^b^

1^c^	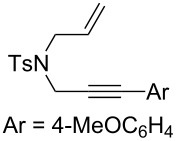	**1c**	17	8	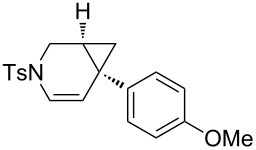	**3c**	77
2^c,d^	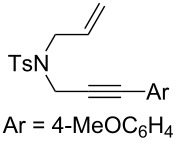	**1c**	17	8	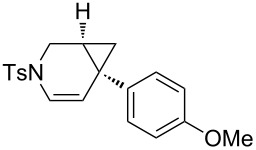	**3c**	89
3^c^	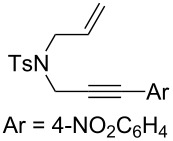	**1d**	24	7	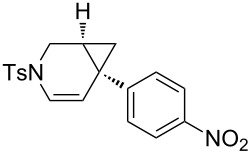	**3d**	35
4^c^	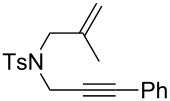	**1e**	16	61	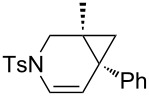	**3e**	13
5^c^	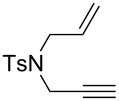	**1b**	16	23	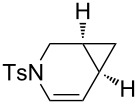	**3b**	22
6	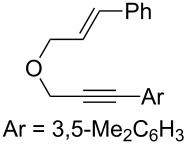	**2b**	3.5	54	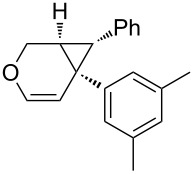	**4b**	93 (+)
7	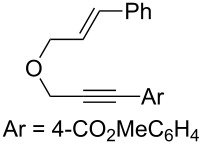	**2c**	15	25	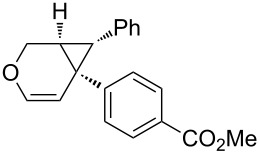	**4c**	94 (−)
8^d^	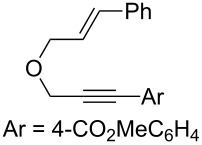	**2c**	15	64	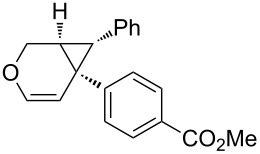	**4c**	94 (−)
9	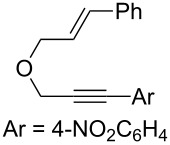	**2d**	15	32	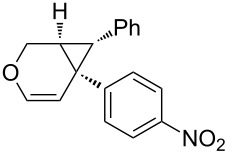	**4d**	96 (−)
10^d^	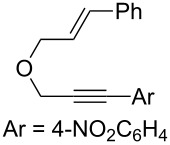	**2d**	15	63	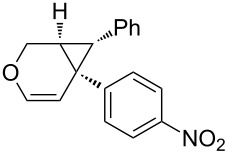	**4d**	98 (−)
11	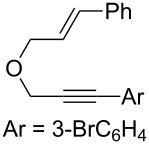	**2e**	30	59	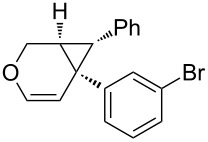	**4e**	95 (−)
12	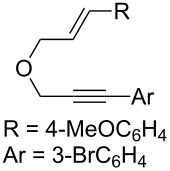	**2f**	1	37	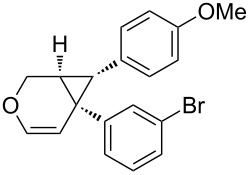	**4f**	95 (−)

^a^Isolated yield, ^b^determined by HPLC, ^c^60 °C, ^d^AgNTf_2_.

**Scheme 4:**
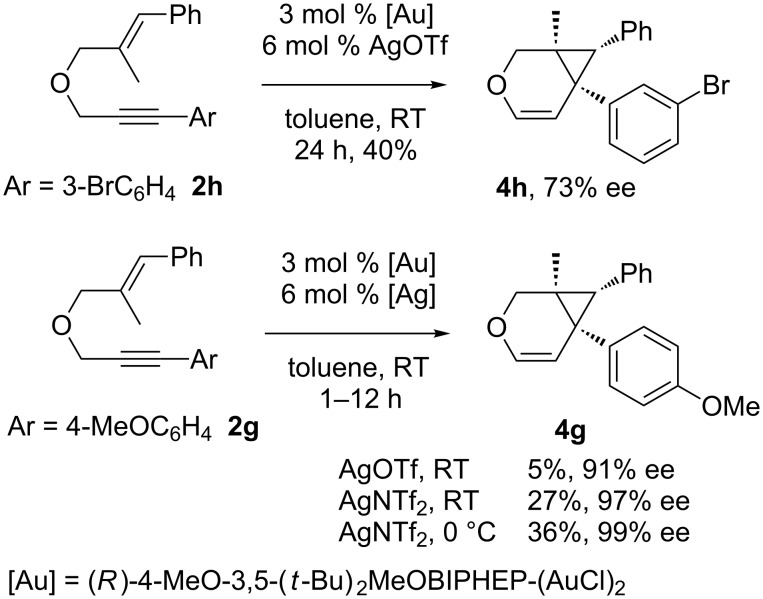
Synthesis of pentasubstituted bicyclic cyclopropanes.

Considering the observed highly stereoselective reactions of oxygen-tethered 1,6-enynes, we decided to study the challenging asymmetric synthesis of pentasubstituted cyclopropyl derivatives [67–70] (Scheme 4). The bicyclic derivative **4h** was obtained in moderate yield and 73% ee. Conducting the reaction at 0 °C and using AgNTf_2_ as a chloride scavenger led to the formation of the alkenyl functionalized derivative **4g** in 36% isolated yield and excellent 99% ee.

## Conclusion

In conclusion, we have contributed to the development of an asymmetric gold-catalyzed cycloisomerization reaction allowing the formation of oxa- and aza-bicyclo[4.1.0]heptene derivatives. The combination of chiral Au(I) complex (*R*)-4-MeO-3,5-(*t*-Bu)_2_-MeOBIPHEP-(AuCl)_2_ associated to silver salts promotes the enantioselective rearrangement of oxygen and nitrogen-tethered 1,6-enynes in toluene at room temperature or 60 °C. The cycloisomerization reactions were found to be highly substrate-dependent as low yield and ee’s were generally obtained in the case of nitrogen-tethered enynes. The enantiomerically enriched functionalized oxabicyclo[4.1.0]heptenes were isolated in moderate yields but with excellent ee values ranging from 73% to 99%. This methodology was successfully applied to the synthesis of pentasubstituted cyclopropyl heterobicycles.

## Experimental

All reactions were carried out under an argon atmosphere. ^1^H NMR and ^13^C NMR were recorded on a Bruker AV 300 instrument. All signals were expressed as ppm (δ) and internally referenced to residual proton solvent signals. Coupling constants (*J*) are reported in Hz and refer to apparent peak multiplicities. Enantiomeric excesses were determined by high pressure liquid chromatography analyses (HPLC) on Waters instruments (Waters 486 detector, 717 autosampler equipped with Daicel Chiralcel OD-H, OJ and Chiralpak IA, AD, λ = 215 nm). Optical rotation measurements were conducted on a Perkin–Elmer 241 polarimeter at 589 nm. Enynes **5** [71], **2a** [72], **2b**–**e** [46], **1a** [73], **1b** [74], **1c**,**d** [75], **1e** [73], and **6** [64] were prepared according to published procedures. ^1^H, ^13^C NMR and mass spectrometry data for compounds **3a**,**b** [23], **3c**,**d** [75], **3e** [76] and **4a**–**e** [46] were described elsewhere.

**(*E*)-1-Bromo-3-(3-(3-(4-methoxyphenyl)allyloxy)prop-1-ynyl)benzene (2f):** CuI (46 mg, 0.1 equiv) and PdCl_2_(PPh_3_)_2_ (86 mg, 0.05 equiv) were placed in a Schlenk tube under argon. Distilled diisopropylamine (3 mL) was added and the reaction mixture was stirred at RT for 5 min. 1-Bromo-3-iodobenzene (0.4 mL, 1.3 equiv) was added and the reaction mixture was stirred for 5 min. Enyne **6**, dissolved in 2 mL of distilled diisopropylamine was added and the reaction mixture stirred for 3 h at RT. After hydrolysis with sat. aq. NH_4_Cl solution, the aqueous phase was extracted with EtOAc. The organic layer was successively washed with sat. aq. NH_4_Cl solution and brine. The organic layer was then dried with MgSO_4_, filtered and the solvents were evaporated under reduced pressure. The crude product was purified by silica gel chromatography (cyclohexane/ethyl acetate 90:10) to give **2f** as a colorless oil (509 mg, 58%). TLC (cyclohexane/ethyl acetate 70:30) *R*_f_ 0.77; ^1^H NMR (300 MHz, CDCl_3_) δ 3.71 (s, 3H), 4.17 (dd, *J* = 6.3, 1.2 Hz, 2H), 4.30 (s, 2H), 6.08 (dt, *J* = 15.9, 6.3 Hz, 1H), 6.52 (d, *J* = 15.9 Hz, 1H), 6.76 (d, *J* = 8.8 Hz, 2H), 7.07 (t, *J* = 7.9 Hz, 1H), 7.16–7.33 (m, 3H), 7.36 (dt, *J* = 8.0, 1.1 Hz, 1H), 7.5 (t, *J* = 1.6 Hz, 1H); ^13^C NMR (75 MHz, CDCl_3_) δ 55.6, 57.9, 71.0, 85.1, 87.1, 114.4 (2C), 122.4, 123.2, 125.1, 128.1 (2C), 129.7, 130.1, 130.7, 132.0, 133.6, 134.9, 159.8.

**(*E*)-1-Methoxy-4-(3-(2-methyl-3-phenylallyloxy)prop-1-ynyl)benzene (2g):** Following the same procedure as for the synthesis of **2f**, in the presence of CuI (103 mg, 0.1 equiv) and PdCl_2_(PPh_3_)_2_ (190 mg, 0.05 equiv), 1-methoxy-4-iodobenzene (1.52 g, 1.2 equiv) in distilled diisopropylamine (10 mL), (*E*)-(2-methyl-3-(prop-2-ynyloxy)prop-1-enyl)benzene [77] (1 g, 1 equiv) was transformed to **2g** (896 mg) in 57% yield. TLC (cyclohexane/ethyl acetate 90:10) *R*_f_ 0.71; ^1^H NMR (300 MHz, CDCl_3_) δ 2.20 (d, *J* = 1.2 Hz, 3H), 4.07 (s, 3H), 4.44 (d, *J* = 0.9 Hz, 2H), 4.66 (s, 2H), 6.83 (s, 1H), 7.11 (m, 2H), 7.68–7.48 (m, 7H); ^13^C NMR (75 MHz, CDCl_3_) δ 15.7, 55.3, 57.8, 76.0, 83.8, 86.2, 113.9, 114.8, 126.5, 127.8, 128.1 (2C), 128.9 (2C), 133.3 (2C), 134.5, 137.4, 159.7.

**(*E*)-1-Bromo-3-(3-(2-methyl-3-phenylallyloxy)prop-1-ynyl)benzene (2h):** Following the same procedure as for the synthesis of **2f**, in the presence of CuI (62 mg, 0.1 equiv) and PdCl_2_(PPh_3_)_2_ (115 mg, 0.05 equiv), 1-bromo-3-iodobenzene (0.53 mL, 1.3 equiv) in distilled diisopropylamine (6.8 mL), (*E*)-(2-methyl-3-(prop-2-ynyloxy)prop-1-enyl)benzene [77] (611 mg, 1 equiv) was transformed to **2h** (851 mg) in 76% yield. TLC (cyclohexane/ethyl acetate 90:10) *R*_f_ 0.63; ^1^H NMR (300 MHz, CDCl_3_) δ 2.08 (d, *J* = 1.3 Hz, 3H), 4.31 (d, *J* = 0.8 Hz, 2H), 4.53 (s, 2H), 6.71 (d, *J* = 1.0 Hz, 1H), 7.30 (t, *J* = 7.8 Hz, 1H), 7.36–7.56 (m, 6H), 7.58 (dt, *J* = 8.0, 0.8 Hz, 1H), 7.75 (t, *J* = 1.6 Hz, 1H); ^13^C NMR (75 MHz, CDCl_3_) δ 16.0, 58.0 (2C), 85.1, 87.1, 122.5, 125.1, 127.0, 128.3, 128.5 (2C), 129.3 (2C), 130.1, 130.7, 132.0, 134.7, 134.9, 137.7.

**General procedure for Au(I)-catalyzed cycloisomerization reactions:** A mixture of L-(AuCl)_2_ (L = (*R*)-4-MeO-3,5-(*t*-Bu)_2_MeOBIPHEP) (3 mol %) and AgOTf (or AgNTf_2_) (6 mol %) in distilled toluene (0.5 M) was stirred under an argon atmosphere at room temperature for 30 min. Enyne (1 equiv) was then added and the mixture stirred until completion of the reaction. The mixture was then filtered through a short pad of silica to eliminate the catalyst (EtOAc) and the solvents were concentrated under reduced pressure. The crude product was purified by silica gel flash chromatography (petroleum ether/ethyl acetate 98:2 to 80:20 v/v) if necessary.

**6-(3-Bromophenyl)-7-(4-methoxyphenyl)-3-oxabicyclo[4.1.0]hept-4-ene (4f):** TLC (cyclohexane/ethyl acetate 80:20) *R*_f_ 0.70; ^1^H NMR (300 MHz, CDCl_3_) δ 2.29 (d, *J* = 5.4 Hz, 1H), 2.65 (d, *J* = 6.0 Hz, 1H), 3.61 (s, 3H), 3.96 (dd, *J* = 10.6, 1.9 Hz, 1H), 4.30 (d, *J* = 10.6 Hz, 1H), 5.21 (d, *J* = 6.0 Hz, 1H), 6.18 (d, *J* = 6.0 Hz, 1H), 6.52–6.65 (m, 4H), 6.86–6.92 (m, 2H), 7.12–7.19 (m, 2H); ^13^C NMR (75 MHz, CDCl_3_) δ 29.9, 30.3, 37.3, 55.5, 61.6, 111.0, 113.7 (2C), 122.5, 128.7, 129.0 (2C), 129.4, 129.9, 130.1, 133.0, 141.2, 142.8, 158.2; HPLC (Chiralpack AD, hexane/propan-2-ol (97:3), flow rate 1.0 mL/min, λ = 215 nm): retention times 7 and 7.5 min, ee 95%; [α]_D_^23^ −18.6 (*c* 1, CHCl_3_).

**6-(4-Methoxyphenyl)-1-methyl-7-phenyl-3-oxabicyclo[4.1.0]hept-4-ene (4g):** TLC (cyclohexane/ethyl acetate 90:10) *R*_f_ 0.66; ^1^H NMR (300 MHz, CDCl_3_) δ 1.18 (s, 3H), 2.84 (s, 1H), 3.73 (d, *J* = 10.4 Hz, 1H), 3.80 (s, 3H), 4.15 (d, *J* = 10.4 Hz, 1H), 5.19 (d, *J* = 5.8, 1 Hz, 1H), 6.21 (d, *J* = 5.8 Hz,1H), 6.80–6.86 (m, 4H), 7.01–7.04 (m, 2H), 7.12–7.15 (m, 3H); ^13^C NMR (75 MHz, CDCl_3_) δ 12.9, 31.6, 35.2, 38.2, 55.2, 67.8, 113.7, 114.5, 125.4, 127.5 (2C), 130.0 (2C), 130.7, 132.1 (2C), 137.5, 140.0, 158.1; HPLC (Chiralcel OJ, hexane/propan-2-ol (99/1), flow rate 1.0 mL/min, λ = 215 nm): retention times 20.3 and 27.1 min, ee 99%; [α]_D_^23^ +26.1 (*c* 1, CHCl_3_).

**6-(3-Bromophenyl)-1-methyl-7-phenyl-3-oxabicyclo[4.1.0]hept-4-ene (4h):** TLC (cyclohexane/ethyl acetate 90:10) *R*_f_ 0.71; ^1^H NMR (300 MHz, CDCl_3_) δ 1.10 (s, 3H), 2.80 (s, 1H), 3.64 (d, *J* = 10.5 Hz, 1H), 4.07 (d, *J* = 10.5 Hz, 1H), 5.09 (d, *J* = 5.8 Hz, 1H), 6.16 (d, *J* = 5.8 Hz, 1H), 6.75 (d, *J* = 2.1 Hz, 1H), 6.78 (d, *J* = 4.0 Hz, 1H), 6.92 (dt, *J* = 7.7, 1.4 Hz, 1H), 7.00–7.20 (m, 5H), 7.29 (dt, *J* = 7.8, 1.2 Hz, 1H); ^13^C NMR (75 MHz, CDCl_3_) δ 14.4, 33.2, 36.5, 39.7, 68.9, 114.6, 123.5, 127.2, 129.0 (2C), 131.1 (2C), 131.2, 131.3 (2C), 135.3, 138.2, 142.0, 142.5; HPLC (Chiralpak IA, hexane/propan-2-ol (99.9:0.1), flow rate 0.5 mL/min, λ = 215 nm): retention times 11.8 and 12.6 min, ee 73%; [α]_D_^23^ +11.7 (*c* 1, CHCl_3_).

## Supporting Information

File 1 Spectral data.
