# Supplementary material for: Asymmetric Au-catalyzed cycloisomerization of 1,6-enynes: An entry to bicyclo[4.1.0]heptene
Source: Beilstein J Org Chem. 2011 Jul 26;7:1021–9. doi: 10.3762/bjoc.7.116 (PMC3169187; doi:10.3762/bjoc.7.116)

Supporting Information

for

Asymmetric Au-catalyzed cycloisomerization of 1,6-enynes: An entry to bicyclo[4.1.0]heptene

Alexandre Pradal, Chung-Meng Chao, Patrick Y. Toullec and Véronique Michelet*

Address: Laboratoire Charles Friedel, UMR 7223, Ecole Nationale Supérieure de Chimie de Paris, Chimie ParisTech, 11 rue P. et M. Curie, F-75231 Paris Cedex 05, France

Email: Véronique Michelet - veronique-michelet@chimie-paristech.fr

* Corresponding author

Spectral data

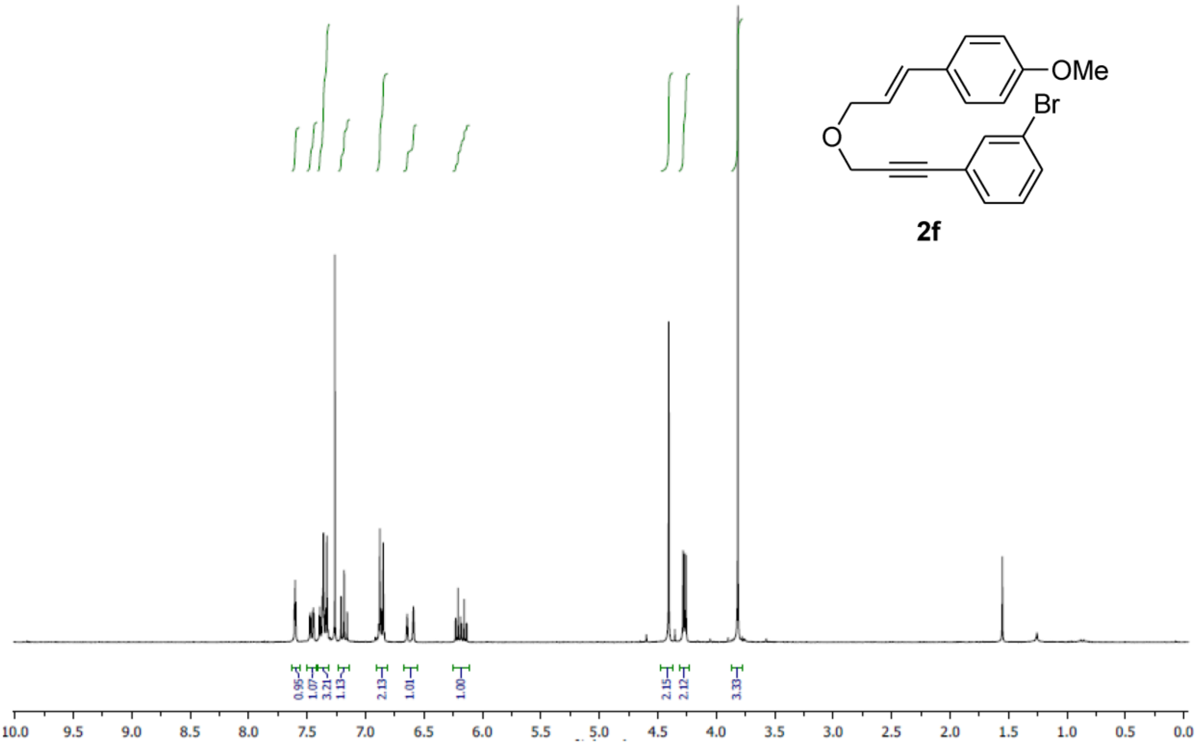

2f

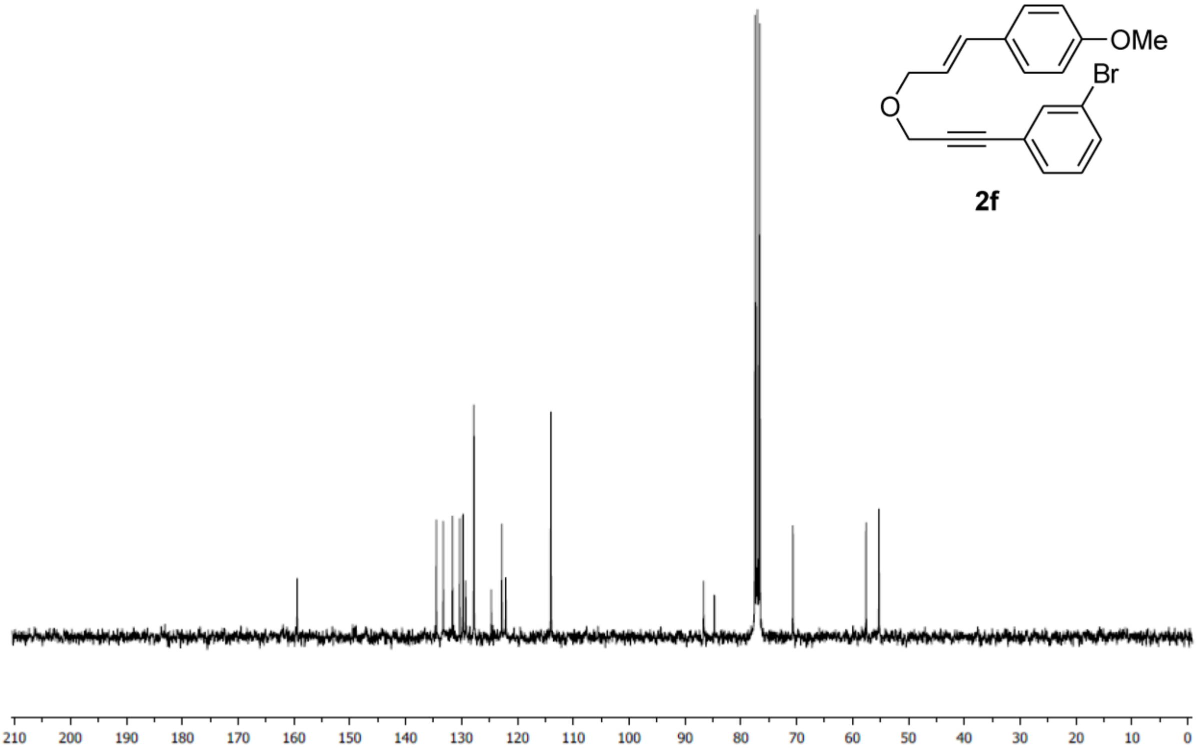
OMe
Br
O
210 200 190 180 170 160 150 140 130 120 110 100 90 80 70 60 50 40 30 20 10 0

2f

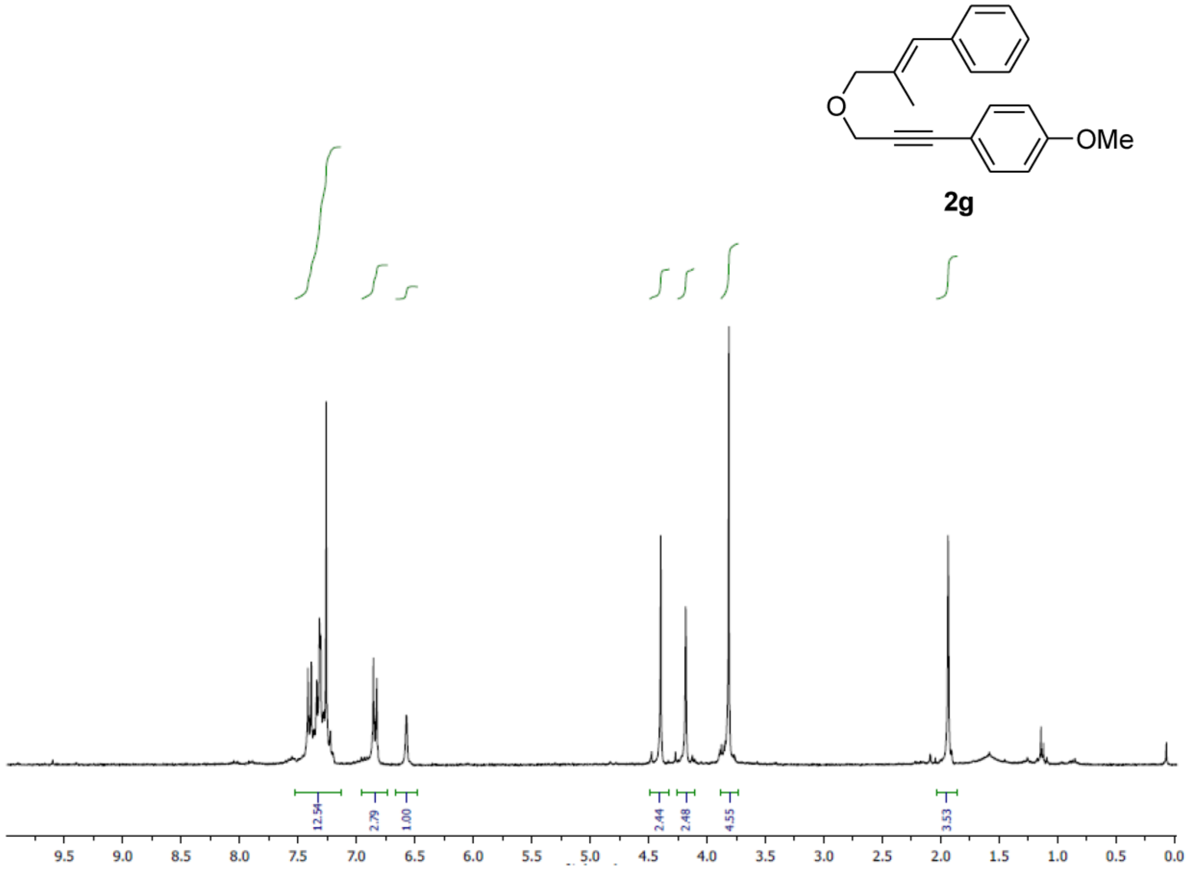
O
OMe
12.54
2.79
1.00
2.44
2.48
4.55
3.53
9.5 9.0 8.5 8.0 7.5 7.0 6.5 6.0 5.5 5.0 4.5 4.0 3.5 3.0 2.5 2.0 1.5 1.0 0.5 0.0

2g

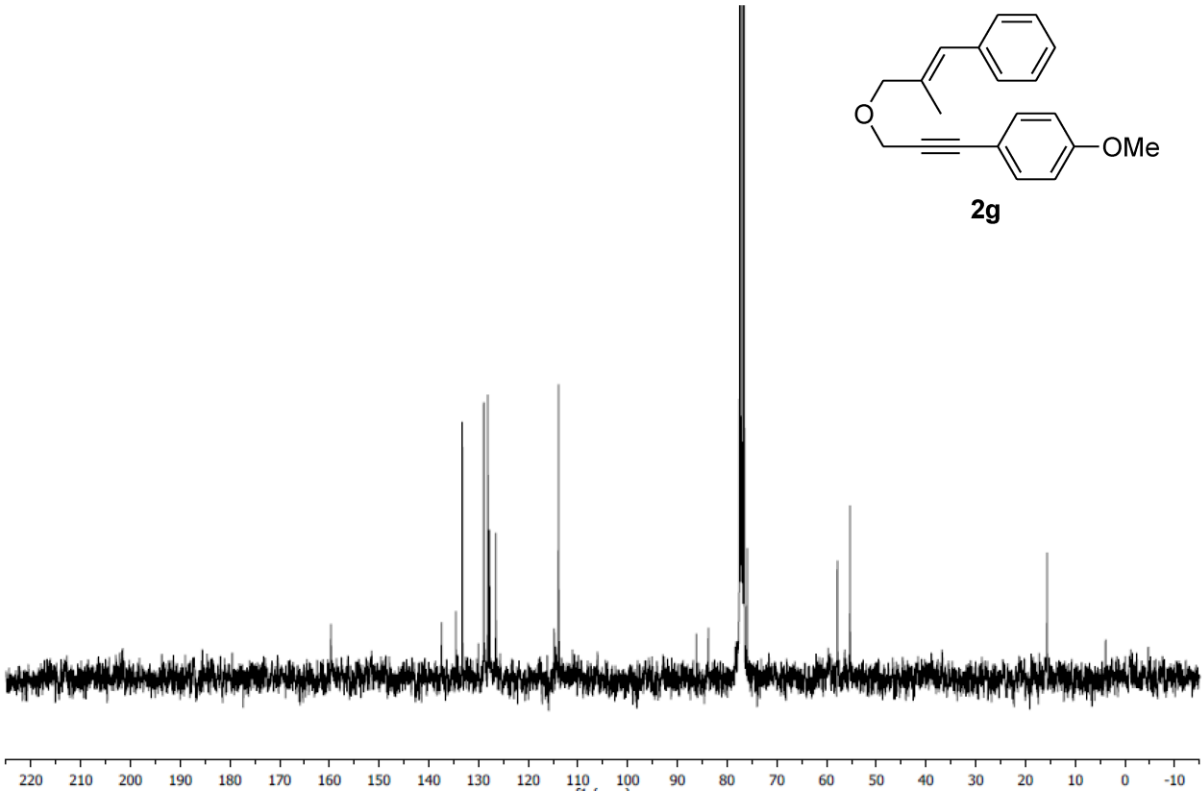
OMe
2g
220 210 200 190 180 170 160 150 140 130 120 110 100 90 80 70 60 50 40 30 20 10 0 -10

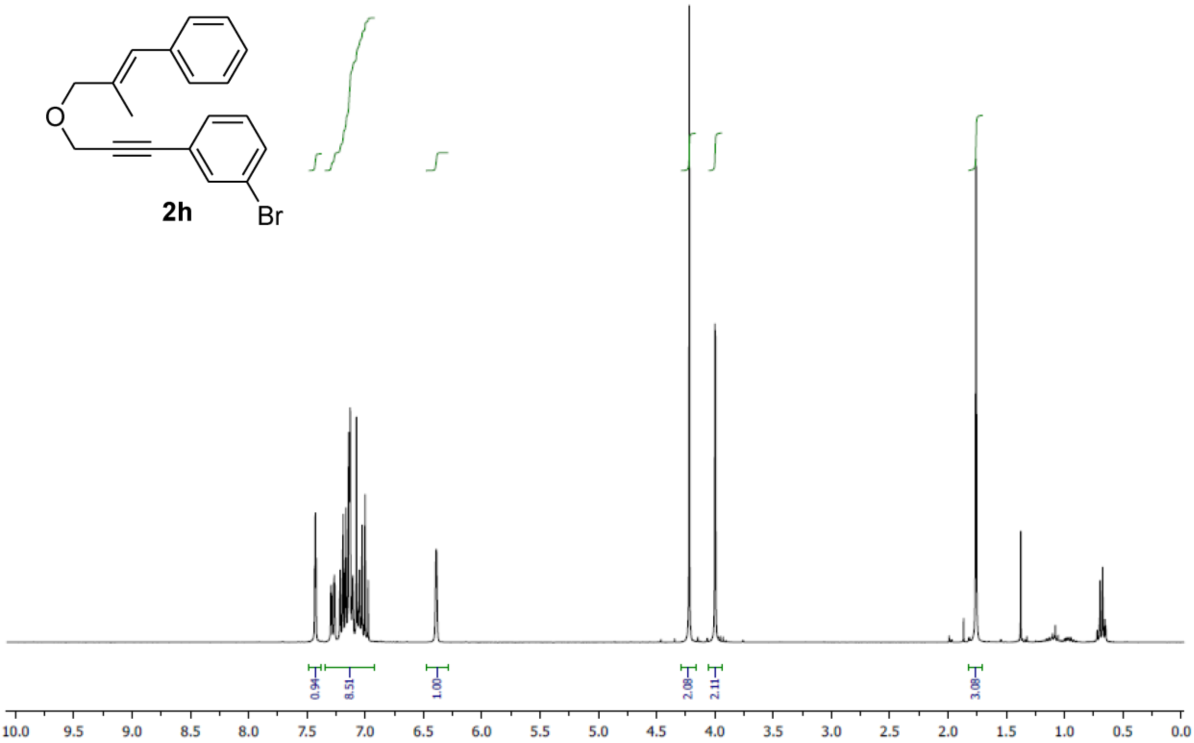
2h
Br
0.94 8.51 1.00 2.08 2.11 3.08
10.0 9.5 9.0 8.5 8.0 7.5 7.0 6.5 6.0 5.5 5.0 4.5 4.0 3.5 3.0 2.5 2.0 1.5 1.0 0.5 0.0

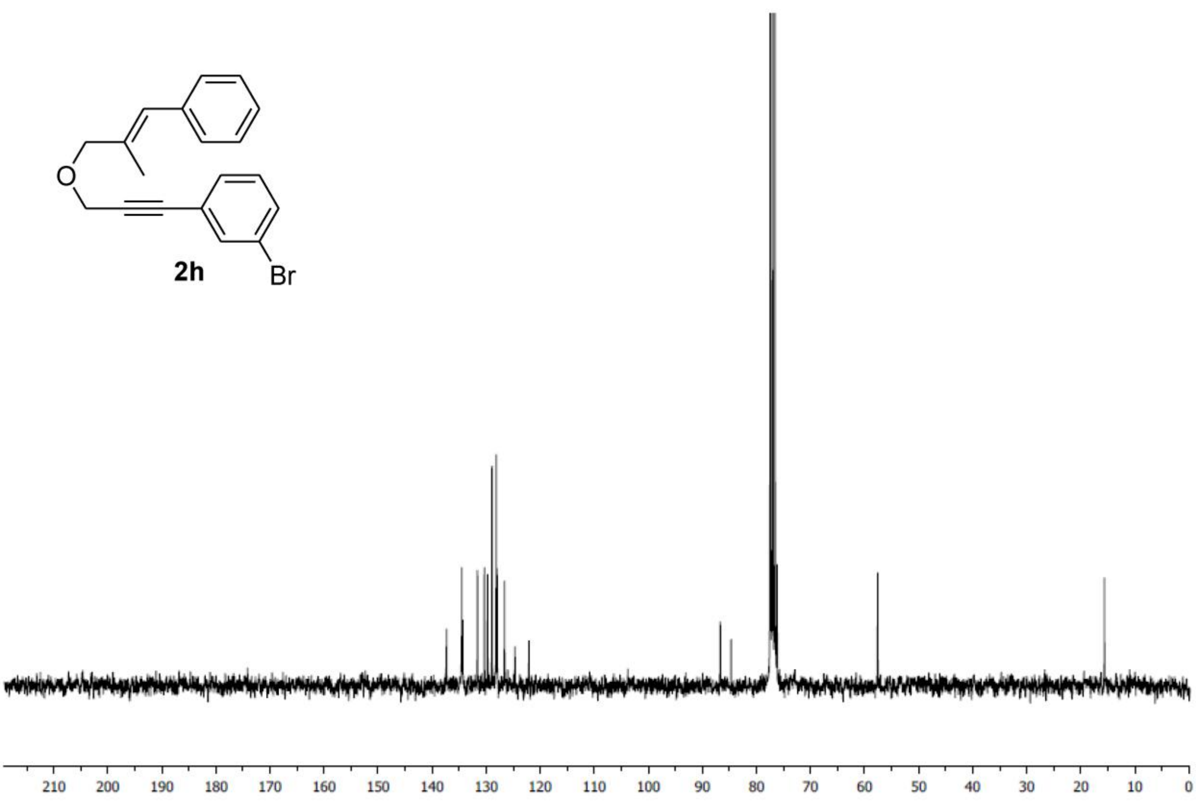
2h
Br
O
210 200 190 180 170 160 150 140 130 120 110 100 90 80 70 60 50 40 30 20 10 0

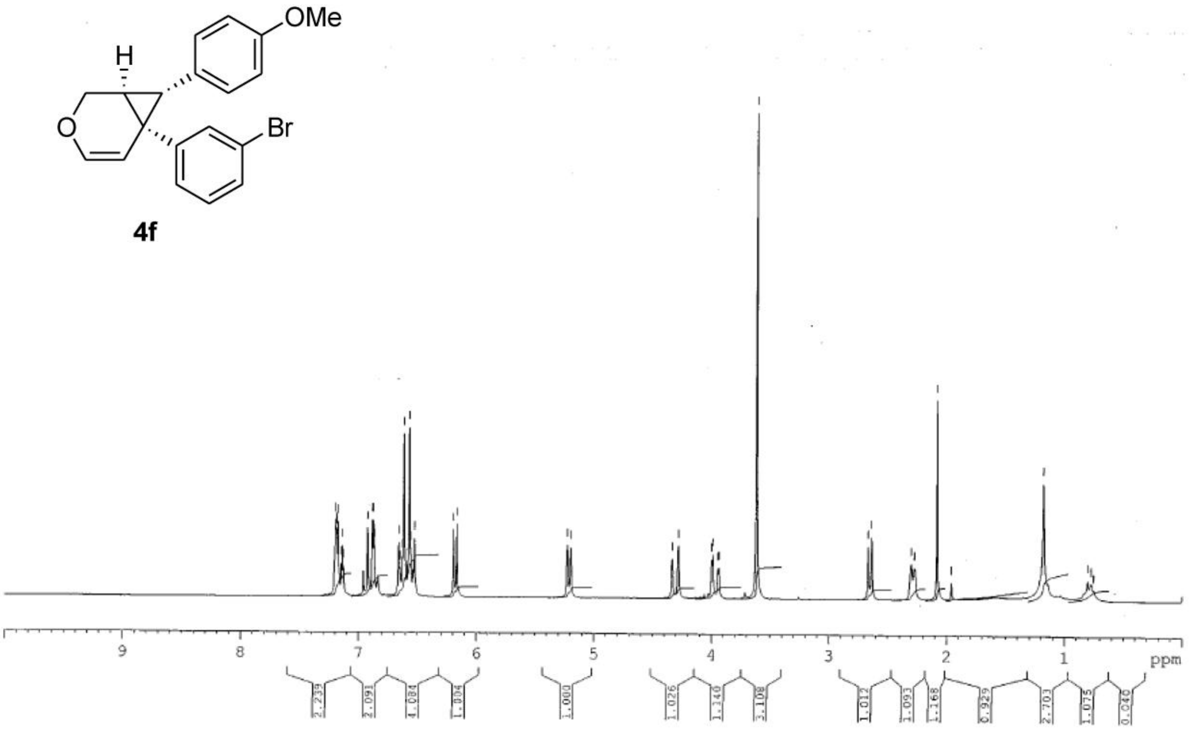
4f
OMe
H
O
Br
9 8 7 6 5 4 3 2 1 ppm

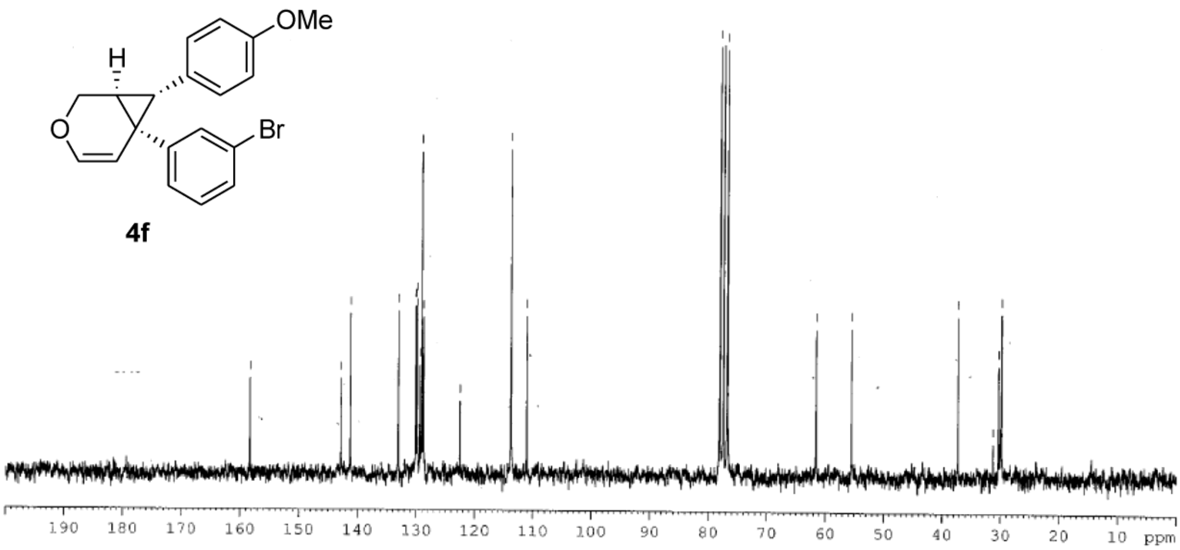

Column : Chiracel AD-H
Eluant : Hex/i-PrOH 97/3
Flow (mL/min) : 1,0

	Retention Time (min)	Area	% Area
1	7,006	319515	2,04
2	7,530	15342711	97,96

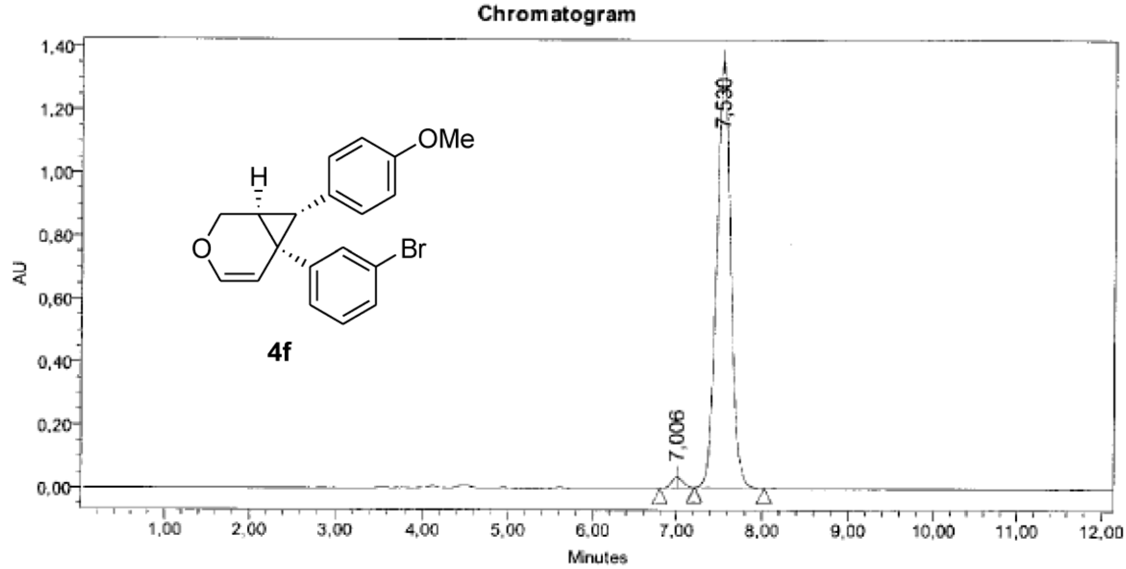

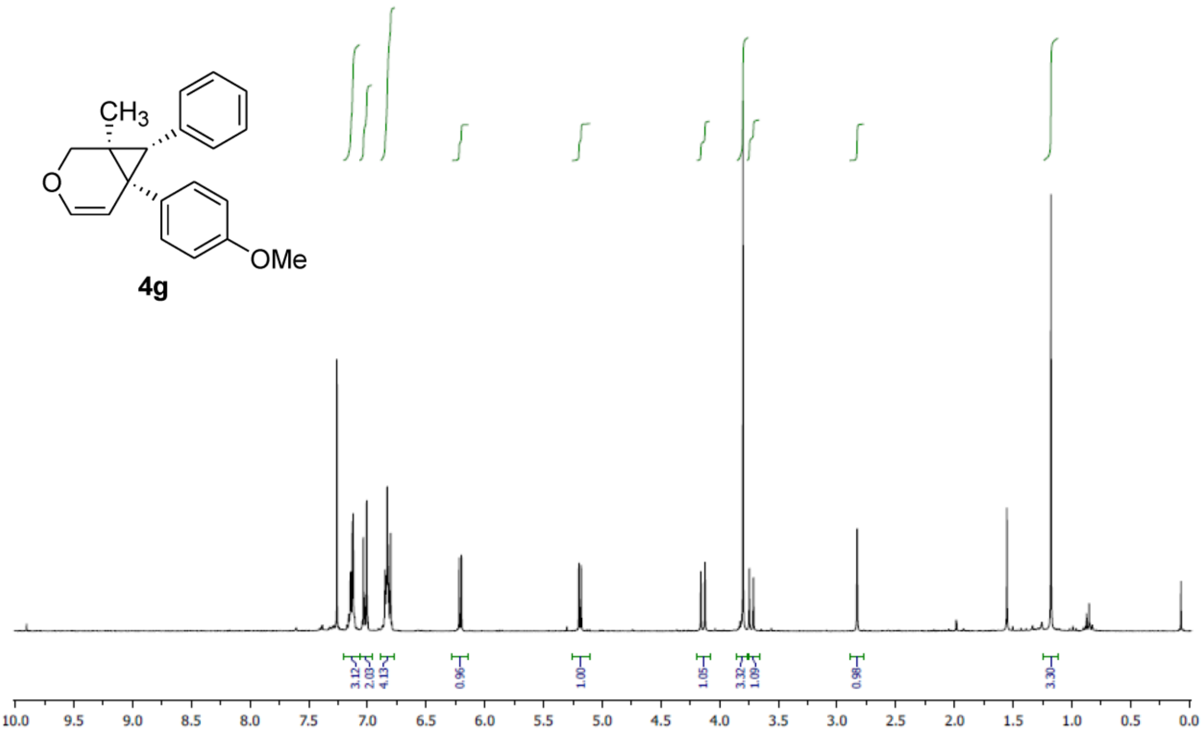
CH3
O
OMe
4g
3.12
2.03
4.13
0.96
1.00
1.05
3.32
1.09
0.98
3.30
10.0
9.5
9.0
8.5
8.0
7.5
7.0
6.5
6.0
5.5
5.0
4.5
4.0
3.5
3.0
2.5
2.0
1.5
1.0
0.5
0.0

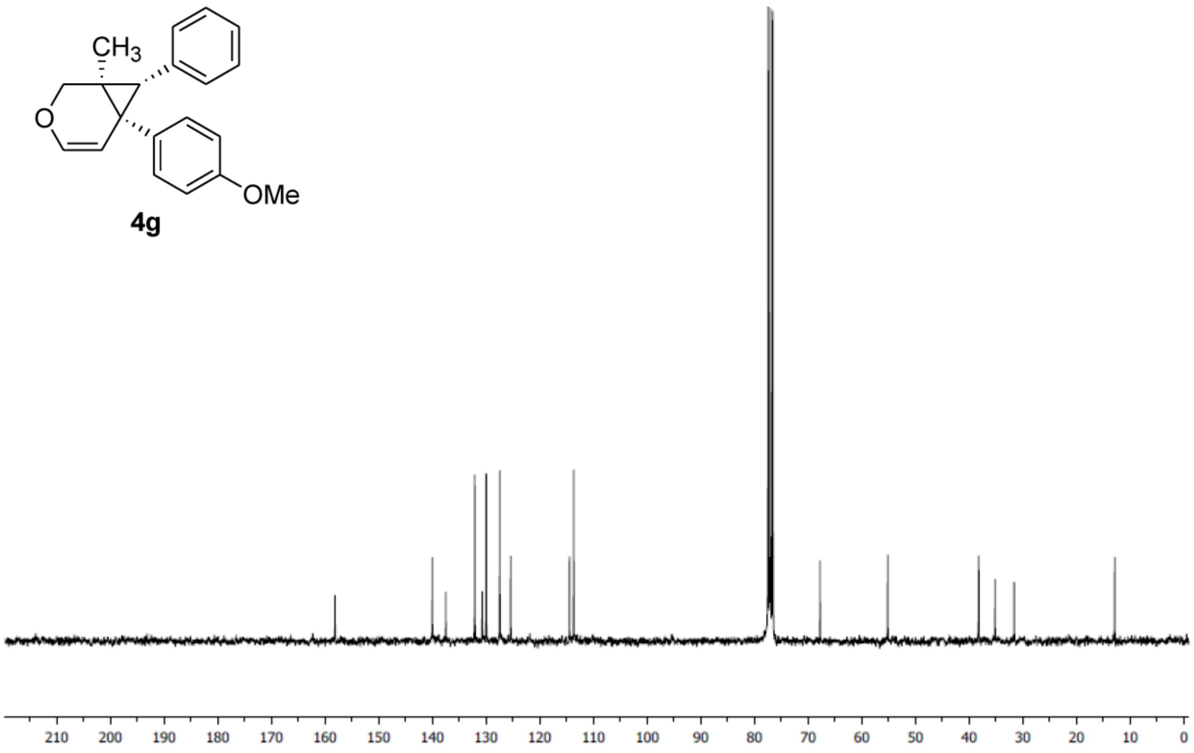
CH3
O
OMe
4g
210
200
190
180
170
160
150
140
130
120
110
100
90
80
70
60
50
40
30
20
10
0

Eluant (Hexane/i-PrOH) : 99/1
Column : Chiracel OJ
Flow : 0.5 mL/min
T° : 20°C

	Retention Time (min)	% Area
1	20.341	0.14
2	27.076	99.86

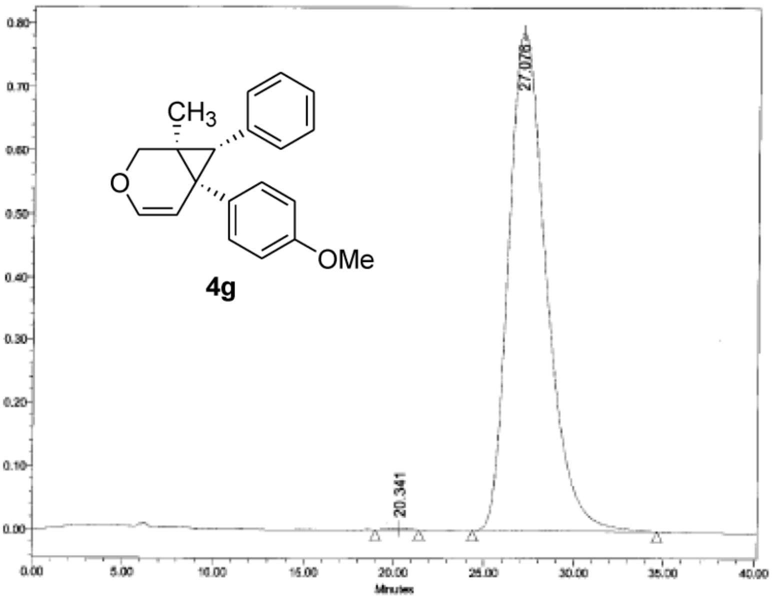

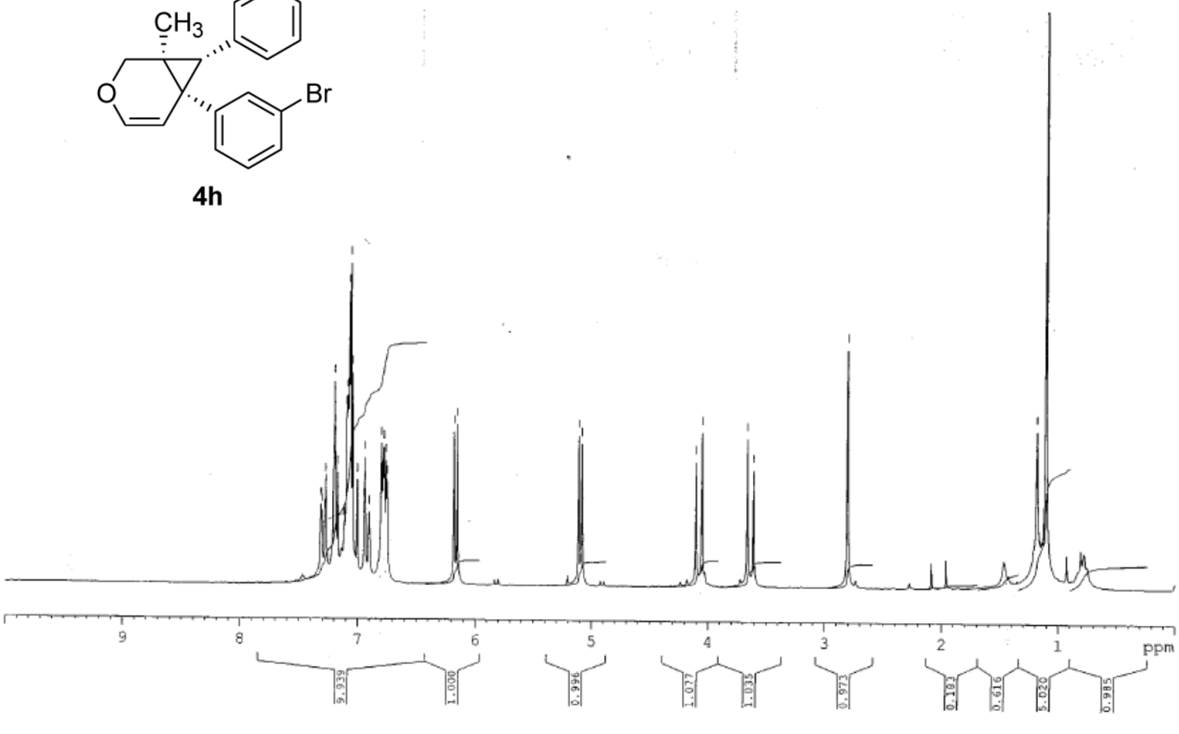

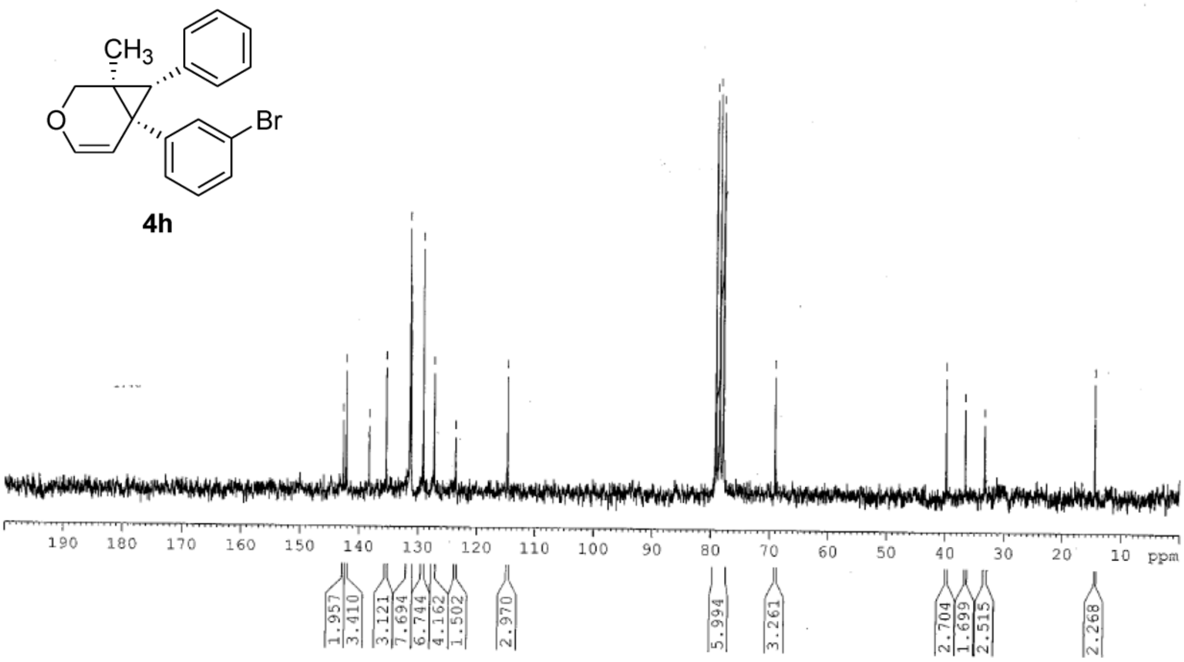

Eluant (Hexane/i-PrOH) : 99.9/0.1
Column : Chiracel IA
Flow : 0.5 mL/min
T° : 20°C

	Retention Time (min)	% Area
1	11.804	13.60
2	12.607	86.40

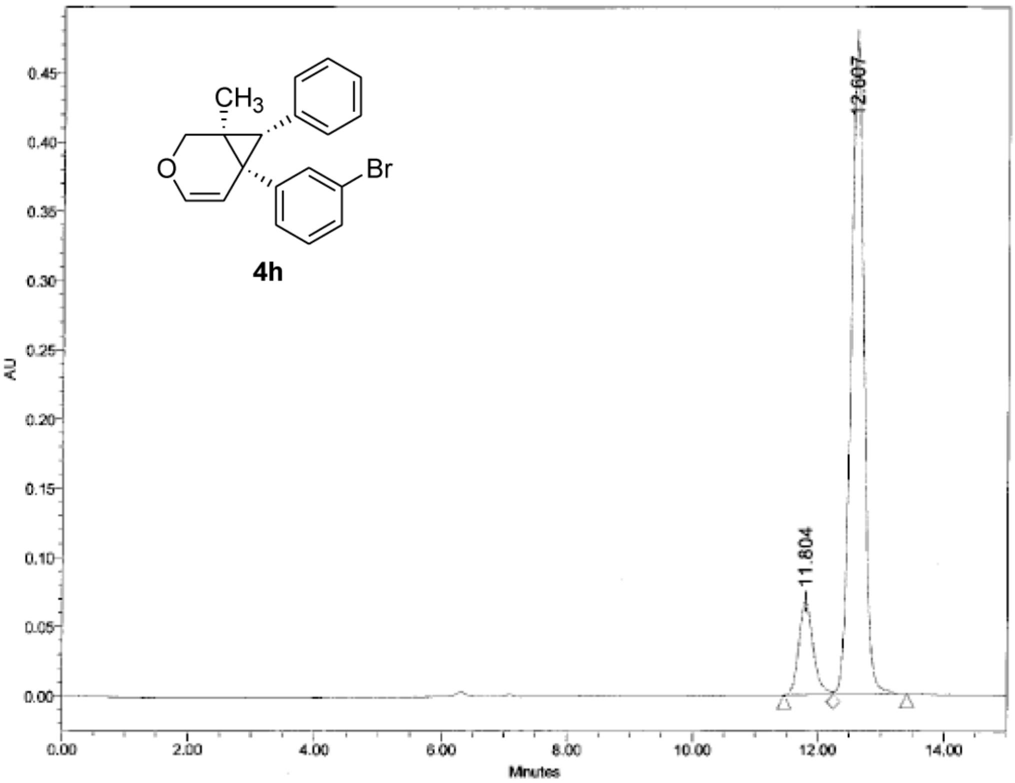

Supplement: File 1 — Spectral data. [file Beilstein_J_Org_Chem-07-1021-s001.pdf]
